# The Mediating Role of Working Hours in the Effect of Surgical and Internal Medicine Residents’ Fear of Malpractice on Defensive Medical Practices

**DOI:** 10.5152/eurasianjmed.2026.251279

**Published:** 2026-02-16

**Authors:** Kamber Kasali, Agah Abdullah Kahramanlar, Mehmet Akif Yılmaz, Habip Burak Özgödek, Eyüp Şenocak

**Affiliations:** 1 Department of Biostatistics, Atatürk University Faculty of Medicine, Erzurum, Türkiye; 2 Anesthesiology Clinical Research Office, Atatürk University, Erzurum, Türkiye; 3 Department of Anesthesiology, Erzurum City Hospital, Erzurum, Türkiye; 4 Department of Anesthesiology and Reanimation, Atatürk University Faculty of Medicine, Erzurum, Türkiye; 5 Department of Orthopaedics and Traumatology, Atatürk University Faculty of Medicine, Erzurum, Türkiye

**Keywords:** Defensive medicine, malpractice, physicians, working hours

## Abstract

**Background:**

The fear of malpractice is a significant factor influencing physicians’ decision-making processes in modern medical practice. This can cause defensive medicine, which involves behaviors such as requesting unnecessary tests and imaging, avoiding high-risk patients, and applying cautious but sometimes medically unnecessary treatments. The aim of this study is to evaluate the effect of surgical and internal medicine residents’ fear of malpractice on defensive medicine attitudes, while examining the mediating role of working hours on this relationship.

**Methods:**

This study is a cross-sectional investigation designed to examine the impact of surgical and internal medicine residents’ fear of malpractice on defensive medical practices. The Fear of Malpractice Scale and the Defensive Medical Practices Attitude Scales were used to collect data. Years of residency, working hours, and opinions on the subject were also included during data collection.

**Results:**

The findings revealed a statistically significant and strong positive correlation between fear of malpractice and defensive medical practices. The total score on the Malpractice Fear Scale showed a strong positive correlation with the total score on the Defensive Medicine Practices Attitude Scale. However, work variables such as average weekly working hours and length of residency do not play a mediating role in the relationship between fear of malpractice and defensive medical practices.

**Conclusion:**

This study supports the notion that fear of malpractice is a central factor influencing physicians’ decision-making processes and increases positive defensive behaviors, such as requesting unnecessary tests, and negative defensive behaviors, such as avoiding high-risk patients.

**Main Points**

- Malpractice fear is the strongest and most direct cause of defensive medical practices among surgical and internal medicine residents.
- Defensive medicine increases in 2 forms: both requesting unnecessary tests (positive) and avoiding high-risk patients.
- Workload variables, such as weekly working hours or duration of residency, do not mediate the effect of malpractice fear on defensive medicine.
- Regulating working hours alone is insufficient to reduce defensive medicine tendencies; the systemic fear of litigation must be addressed.
- The finding that the vast majority of participants (92%) lack formal malpractice law training suggests that a fundamental knowledge gap contributes to defensive practices.

## Introduction

The fear of malpractice is a significant factor influencing physicians’ decision-making processes in modern medical practice. Surgical and internal medicine residents may resort to defensive medicine practices in their clinical work to ensure patient safety and avoid legal risks.^1^ Defensive medicine involves behaviors such as requesting unnecessary tests and imaging, avoiding high-risk patients, and applying cautious but sometimes medically unnecessary treatments.^2^

Research shows that assistants working in surgical specialties practice defensive medicine at a higher level than their colleagues in internal medicine.^3^ The prevalence of defensive medicine practices stems from the high workload, patient responsibility, and legal pressures faced by those working in surgical specialties in particular.^4^ Furthermore, some studies reveal that students are exposed to defensive medicine practices even during their medical school education, and that these practices shape their clinical decision-making processes after graduation.^5^^-^^7^

The fear of malpractice has been found to affect physicians’ decision-making processes not only at the individual level but also across the healthcare system as a whole.^8^ For example, physicians ordering unnecessary tests increase the costs of the healthcare system and, in some cases, may create unnecessary risks for patients.^9^ Furthermore, it has been shown that defensive medical attitudes lead to burnout syndrome in physicians, with long working hours and excessive workloads exacerbating this situation.^1^

On the other hand, some studies show that fear of malpractice diminishes over time and that physicians are able to make more rational decisions as their clinical experience increases.^10^ However, it has been determined that young assistant physicians practice more defensively at the beginning of their professional careers and that these fears diminish over time.^11^

The aim of this study is to evaluate the effect of surgical and internal medicine residents’ fear of malpractice on their defensive medicine attitudes, while examining the mediating role of working hours on this relationship. By investigating how long working hours interact with fear of malpractice and how this is reflected in defensive medicine practices, important implications for health policies will be presented.

## Material and Methods

### Study Design and Participants

This study is a cross-sectional investigation designed to examine the impact of surgical and internal medicine residents’ fear of malpractice on defensive medical practices. Data collection utilized the Fear of Malpractice Scale and the Defensive Medical Practices Attitude Scale. The questionnaire was created using Google Forms and randomly distributed to surgical and internal medicine residents. The first question in the questionnaire was “I voluntarily participate in this study” and only the responses of participants who accepted this question were considered. Informed consent was obtained from all participants. In addition to the study, questions such as years of residency, working hours, and opinions on the subject were also included. Informed consent was obtained from all participants.

### Data Collection Tools

#### Malpractice Fear Scale

This scale is a valid and reliable tool used to measure physicians’ levels of malpractice fear. The Malpractice Fear Scale was developed by Katz et al (2005).^12^ When first developed, it contained 6 items, and its original structure was determined to be unidimensional. The scale was adapted into Turkish by Uğur Uğrak and Oğuz Işık (2020).^13^ Cronbach’s alpha reliability coefficient was found to be 0.860, and the composite reliability coefficient was 0.858. As the scale is unidimensional, it is not divided into subscales. The total score on the scale ranges from 6 to 30, with higher scores indicating greater fear of malpractice.

#### Defensive Medicine Practices Attitude Scale

This scale was used to determine the extent to which physicians resort to defensive medicine practices. This scale was developed by Aysel Başer, Mukadder İnci Başer Kolcu, Giray Kolcu, and Umut Gök Balcı (2014).^14^ As a result of factor analysis, the scale consists of 3 sub-dimensions: Positive Defensive Medical Practices (items 1-9), Negative Defensive Medical Practices (items 10-14), and Knowledge Level (items 15-18). Cronbach’s alpha reliability coefficients were found to be 0.853 for the general scale, 0.685 for Positive Defensive Medical Practices, and 0.918 for Negative Defensive Medical Practices. The sub-dimensions can be evaluated separately, and the total score can also be calculated.

#### Sample Size

The sample size was determined using the Raosoft sample size calculator (Raosoft Inc., Seattle, WA, USA) with a 95% CI, a 50% response rate, and a 5% margin of error, indicating that 255 participants were required. A total of 264 individuals participated in the study.

### Statistical Analysis

The data were presented in terms of mean, standard deviation, median, minimum, maximum, percentage, and frequency. The normality of distribution for continuous variables was assessed using the Shapiro–Wilk test, Kolmogorov–Smirnov test, skewness, and kurtosis (with Lilliefors correction). For comparisons between quantitative variables, Pearson and Spearman correlation tests were used, depending on the normality of distribution. For comparisons between 2 independent groups, the Independent Samples *t*-test was used if the normality assumption was met, and the Mann–Whitney *U*-test was used if it was not. Analysis of Covariance (ANCOVA) was used in multiple comparisons to examine the effects of co-factors on the dependent variable. The significance of indirect effects in the model was used to determine the relationships between the scales tested using the structural equation modeling bootstrapping method. Working hours and residency duration were considered as mediators because both factors are known to modulate the relationship between occupational stressors and professional well-being among physicians (e.g., extended hours and prolonged training increase strain and reduce satisfaction). Multivariate normality was assessed using Mardia’s test, which evaluates skewness and kurtosis and was widely used in Structural Equation Model (SEM) analyses. To address potential deviations from normality, bootstrapping with 1000 resamples was applied. The path coefficients (*β*) of the model were calculated. All analyses were performed using IBM SPSS 20 and JAMOVI 2.2.2 statistical software. Statistical significance was set at *P* < .05.

### Ethical Approval

The Non-Interventional Ethics Committee of Atatürk University Faculty of Medicine has determined that the study is ethically acceptable in its decision dated January 31, 2025, and numbered B.30.2.ATA.0.01.00/55.

## Results

Participants in the study were 55.7% men and 44.3% women (Table 1).

Participants’ 53.8% are married, 44.7% are single, and 1.5% are divorced. 58% of employees work in internal medicine, while 42% work in surgical sciences. The most represented departments are anesthesiology and resuscitation (17.6%), family medicine (13.4%), and dermatology and venereal diseases (8%). 93.1% of participants work in university hospitals, 6.1% in teaching and research hospitals, 0.4% in state hospitals, and 0.4% in private hospitals. Within the last year, 12.2% of participants stated that they had faced allegations of malpractice, while 87.8% stated that they had not experienced such a situation. Furthermore, only 8% of participants stated that they had received training on malpractice law and legal processes, while 92% stated that they had not received such training (Table 1).

The average age of participants is 30.7 ± 5.22. The average duration of assistantship is 33.4 ± 18.76 months. The average weekly working hours are 52.5 ± 18.00 hours.

The mean score on the malpractice fear scale was 3.83 ± 0.89, with a Cronbach’s α value of 0.908 and a McDonald’s ω value of 0.910. The total score for the defensive medicine practices attitude scale was 3.35 ± 0.78, with Cronbach’s α found to be 0.923 and McDonald’s ω 0.930. The positive defensive medical practices subscale was calculated as 3.48 ± 0.83 (α = 0.903, ω = 0.905), while the negative defensive medical practices subscale was calculated as 3.38 ± 1.00 (α = 0.904, ω = 0.906). The knowledge level scale had a mean of 3.04 ± 0.90, with internal consistency coefficients of Cronbach’s α = 0.602 and McDonald’s ω = 0.656 (Table 2).

Within the Malpractice Fear Scale, the highest item-total correlation is observed in Item 5 (0.808), which contributes most strongly to the scale. The lowest contribution is seen in Item 1 (0.594). Within the Defensive Medical Practices Attitude Scale, the highest item-total correlations are found in Item 3 (0.775) and Item 12 (0.728); these items are the items that contribute most to the reliability of the scale. The lowest contribution is found in Item 15 (0.206), indicating the weakest item in the scale (Table 2).

In the comparison between surgical and internal sciences, the mean ages were found to be similar, and no statistically significant difference was observed (*P* = .965) (Table 3). The length of residency was longer in surgical sciences, and this difference was significant (surgical: 36.00 ± 19.71 months; internal: 31.50 ± 17.86 months; *P* = .047). The average weekly working hours were also significantly higher in surgical sciences (surgical: 59.00 ± 16.23 hours; internal: 48.10 ± 19.57 hours; *P* < .001). Although the malpractice fear score was slightly higher in internal medicine, the difference was not statistically significant (*P* = .075). No significant difference was found between the 2 groups in terms of positive and negative defensive medicine practices, knowledge level, and total defensive medicine attitude scale scores (*P* > .05). These results indicate that residents working in the surgical field have longer working hours and a more intensive weekly work schedule (Table 3).

When comparing the gender distribution between surgical and internal medicine departments, no statistically significant difference was found in terms of male and female ratios (χ^2^ = 0.464, *P* = .496). This result indicates that the gender distribution is similar across department types (Table 4).

Positive and significant correlations were found between the total score on the malpractice fear scale and other variables. Fear of malpractice showed a strong positive relationship with positive defensive medical practices (*r* = 0.69), a moderate positive relationship with negative defensive medical practices (*r* = 0.574), and a lower but still significant positive relationship with knowledge level (*r* = 0.449). Furthermore, a strong relationship was found with the total score of the defensive medicine practices attitude scale (*r* = 0.695). All correlations were statistically significant (*P* < .001). These findings indicate that fear of malpractice tends to increase defensive medicine behaviors and knowledge level (Table 5).

According to the results of the structural equation model, fear of malpractice has a direct and significant effect on defensive medical practices and knowledge level. Fear of malpractice significantly increases the attitude score towards defensive medical practices (*β* = 0.695, *P* < .001). This relationship applies to both positive and negative defensive medical practices. In positive practices, *β* = 0.687 (*P* < .001), while in negative practices, *β* = 0.576 (*P* < .001). Furthermore, fear of malpractice significantly increases the level of knowledge (*β* = 0.452, *P* < .001). However, none of these effects occur indirectly through the length of residency or average weekly working hours. Indirect effects were not statistically significant in any of the models (*P* > .05). These findings indicate that fear of malpractice directly increases defensive behavior and knowledge levels; however, variables such as length of residency and working hours do not play a mediating role in this relationship (Table 6).

**Figure 1.**
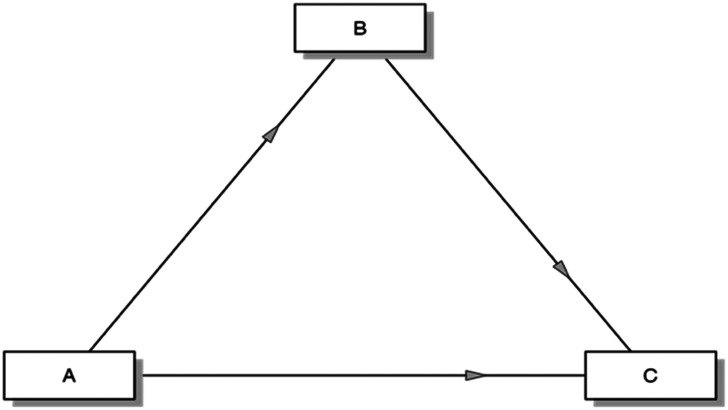
Path model.

The path model is shown in the Figure 1. In the model, A is the independent variable, C is the dependent variable, and B is the mediating variable (Figure 1).

## Discussion

This study aimed to examine the effect of surgical and internal medicine residents’ fear of malpractice on their defensive medical practices and to evaluate the mediating role of their working hours in this relationship. The findings revealed a statistically significant and strong positive correlation between fear of malpractice and defensive medical practices. The total score on the Malpractice Fear Scale showed a strong positive correlation with the total score on the Defensive Medicine Practices Attitude Scale (*r* = 0.695, *P* < .001). This relationship is consistent with the findings of previous studies and supports the notion that fear of malpractice is a central factor influencing physicians’ decision-making processes. ^2^^,^^8^^,^^12^

The fact that fear of malpractice showed a significant correlation with both positive (*r* = 0.69) and negative defensive medical practices (*r* = 0.574) indicates that fear directs physicians towards behaviors such as requesting unnecessary tests (positive defensive medicine) and avoiding high-risk patients (negative defensive medicine). Structural equation modeling analyses confirm that fear of malpractice has a direct and significant enhancing effect on defensive medical practices and knowledge level (*β* = 0.695 for defensive medicine total score, *P* < .001).

Reviewing the literature, Goetz et al^15^ evaluated general practitioners’ actions, their fear of legal consequences, and factors affecting the risk of error and litigation using a questionnaire containing various questions and scored them from 1 to 6. As a result of these surveys, nearly half of the participants stated that their medical practices would be affected by legal consequences to a high or very high degree (48%). Thus, similar to the study, it was shown that defensive medical practices are associated with legal fears. However, unlike this study, Goetz and colleagues classified participants’ fears and factors affecting litigation risk as very low, medium, and very high, and did not use scales constructed as did this study. In this study, participants indicated that the most important reason for defensive behavior was to protect themselves legally.^15^ The use of validated scales to measure defensive behavior in this study is an aspect that makes it superior to others.

Arafa et al^16^ evaluated defensive medical practices using a 6-question survey in their study and, based on this, created a defensive medicine score for each participant. They also evaluated the rate of defensive medical practices among participants according to demographic factors and found that defensive medical approaches were more common among males and younger individuals.^16^ However, unlike this study, the frequency of defensive medical practices did not vary by gender in the study. Furthermore, while the study found no significant difference in defensive medical practices between surgical and internal medicine departments, this study identified obstetrics and gynecology, orthopedics, and plastic surgery as the departments most likely to engage in defensive medical practices, all of which are surgical specialties. Similarly, they have shown that defensive practices are also associated with malpractice claims and, in addition, physical violence in the workplace.^16^

In a recent study by Andersen et al^17^ family physicians’ consultation requests were evaluated, and their defensive medical approaches were examined. In this study, the most common defensive actions reported were unnecessary blood tests and tests that could be performed at the patient’s bedside. The most common reasons for defensive behavior were the patient’s own influence and the fear of missing a serious illness. Furthermore, in the study conducted by Assefa and colleagues,^18^ the most common defensive behavior was avoiding high-risk procedures, followed by requesting unnecessary blood tests; and the most important reason for defensive behavior was stated as fear of being sued.^18^ In this study, the most common defensive behaviors were requesting more consultations to prevent legal issues and avoiding treatment protocols with high complication rates, like Assefa’s study.

On the other hand, previous studies have suggested that working hours (specifically residency duration and average weekly working hours) may play a mediating role in the relationship between fear of malpractice and defensive medical practices.^19^ However, in the present study, the indirect effect analyses did not reveal a statistically significant mediation. The indirect effects mediated by average weekly working hours or duration of residency were found to be insignificant across all models (*P* > .05). This finding indicates that fear of malpractice directly influences physicians’ defensive behaviors, whereas neither the intensity of residents’ workload nor the length of their professional experience significantly mediates this direct relationship.

In the interdepartmental comparison, it was determined that assistants working in surgical sciences had both a longer residency period (36.00 ± 19.71 months) and a significantly higher average weekly working hours (59.00 ± 16.23 hours) compared to those working in internal sciences (*P* < .001). This result supports the literature on high workload and legal pressure in surgical specialties.^20^ However, no statistically significant difference was found between surgical and internal medicine specialties in terms of malpractice fear total score and defensive medicine practices total score (*P* > .05). This suggests that residents in both specialties may be under a similar level of malpractice pressure.

Furthermore, the fact that the vast majority of participants (92%) stated that they had not received training on malpractice law and legal processes, when considered alongside the positive correlation between high levels of malpractice fear and knowledge levels, indicates that the lack of training in this area may play a role in the proliferation of defensive practices.

### Limitations

While the findings of this study offer significant contributions, they also have some methodological and structural limitations. First, the study employed a cross-sectional design, which only assesses the relationship between variables over a specific time period. Therefore, the findings do not allow for causal relationships; they only allow for correlation-based interpretations. Longitudinal or experimental designs could more robustly test the direction of change in fear of malpractice over time and its impact on defensive medical behaviors. Data were collected through self-reports. In areas of high legal and ethical sensitivity, such as defensive medicine, self-reported measures carry a risk of measurement bias. The sample was obtained from a single country and a limited institutional setting (predominantly university hospitals). The fact that 93.1% of the participants worked in university hospitals means that the experiences of residents working in different healthcare institutions were not adequately reflected in the model. This may limit the generalizability of the findings, particularly to private hospitals or low-resource institutions. Furthermore, in the mediation models evaluated in this study, only duration of service and years of residency were examined as mediating variables. However, the literature reports that defensive medical behaviors are associated with factors such as job stress, burnout, organizational culture, specialty risk, level of consultant supervision, or prior litigation experience. Therefore, potential psychosocial and organizational variables not included in the model may account for some of the unexplained variance.

## Results

This study conclusively demonstrates that fear of malpractice directly and significantly increases defensive medical practices among surgical and internal medicine residents (*β* = 0.695, *P* < .001). Fear of malpractice increases both positive defensive behaviors, such as requesting unnecessary tests, and negative defensive behaviors, such as avoiding high-risk patients. However, work variables such as average weekly working hours and length of residency do not play a mediating role in the relationship between fear of malpractice and defensive medical practices. This suggests that simply regulating working hours may not be sufficient to reduce defensive medicine tendencies; rather, it is necessary to focus on the factors that directly cause fear of malpractice and the lack of knowledge about legal processes. The study findings emphasize the urgent need for comprehensive training programs on malpractice law and legal processes, particularly for junior doctors. It is important to provide training on legal responsibilities rather than reforms focused solely on working hours, so it will be beneficial in reducing the fear of malpractice and defensive behaviors.

## Figures and Tables

**Table 1.** The Result of Demographic Data

		**N**	**N (%)**
Gender	Male	146	55.70
	Female	116	44.30
Marital status	Married	141	53.80
	Single	117	44.70
	Divorced	4	1.50
Department	Surgical sciences	110	42.00
	Internal sciences	152	58.00
Specialty field	Anesthesiology and reanimation	46	17.60
	Family medicine	35	13.40
	Dermatology	21	8.00
	Obstetrics and gynecology	20	7.60
	Emergency medicine	16	6.10
	Physical medicine and rehabilitation	16	6.10
	Cardiology	16	6.10
	Pulmonary diseases	14	5.30
	Otorhinolaryngology	12	4.60
	Infectious diseases	8	3.10
	Ophthalmology	8	3.10
	Psychiatry	7	2.70
	Child and adolescent psychiatry	7	2.70
	Radiology	6	2.30
	Urology	5	1.90
	Cardiovascular surgery	4	1.50
	Neurology	4	1.50
	Orthopedics and traumatology	4	1.50
	Internal medicine	3	1.10
	Pathology	2	0.80
	Pediatric surgery	2	0.80
	Pediatrics	2	0.80
	Neurosurgery	1	0.40
	General surgery	1	0.40
	Thoracic surgery	1	0.40
	Public health	1	0.40
Type of institution	University hospital	244	93.10
	Training and research hospital	16	6.10
	State hospital	1	0.40
	Private hospital	1	0.40
Encountered a malpractice claim in the past year?	Yes	32	12.20
	No	230	87.80
Received training on malpractice law and legal procedures?	Yes	21	8.00
	No	241	92.00

**Table 2.** Item Reliability Statistics

Scales		Item	Items	Mean	±SD	If Item Dropped		
						Item-rest Correlation	Cronbach’s *α*	McDonald’s *ω*
Malpractice Fear Scale		Item 1	I have had to make significant changes in my professional practices due to legal developments related to healthcare delivery.	3.19	±1.09	0.594	0.913	0.915
		Item 2	I am concerned that I might be involved in a malpractice lawsuit within the next 10 years.	3.86	±1.08	0.75	0.891	0.896
		Item 3	I feel pressured in my daily professional practice due to the threat of malpractice lawsuits.	3.83	±1.12	0.783	0.886	0.891
		Item 4	I sometimes request certain tests and consultations solely to avoid malpractice.	4.01	±1.07	0.795	0.884	0.887
		Item 5	I occasionally seek additional expert opinions specifically to reduce the risk of being sued.	4.06	±1.02	0.808	0.883	0.885
		Item 6	Relying on clinical judgment rather than technology in diagnosis has become increasingly risky from a medicolegal perspective.	4.05	±1.04	0.75	0.891	0.894
Defensive Medicine Attitude Scale	Positive Defensive Medicine Practices	Item 1	I order diagnostic tests for my patients beyond what is necessary in order to avoid legal issues.	3.23	±1.15	0.659	0.918	0.925
		Item 2	I prescribe most of the medications within indications to avoid potential legal problems.	3.23	±1.08	0.632	0.919	0.926
		Item 3	I request more consultations for possible complications in my patients to avoid legal problems.	3.62	±1.08	0.775	0.915	0.922
		Item 4	I hospitalize patients for non-medical reasons (e.g., social indications) to avoid legal problems.	2.85	±1.13	0.559	0.92	0.928
		Item 5	I use imaging techniques more frequently to avoid legal problems.	3.58	±1.14	0.75	0.916	0.923
		Item 6	I explain medical procedures to my patients in greater detail to avoid legal problems.	3.79	±1.12	0.724	0.916	0.924
		Item 7	I spend more time with my patients to avoid legal problems.	3.26	±1.16	0.547	0.92	0.928
		Item 8	I keep medical records more thoroughly to avoid legal problems.	3.82	±1.06	0.647	0.918	0.926
		Item 9	I place greater emphasis on obtaining informed consent forms to avoid legal problems.	3.92	±1.07	0.655	0.918	0.925
	Negative Defensive Medicine Practices	Item 10	I avoid patients who are more likely to file lawsuits.	3.38	±1.2	0.706	0.917	0.924
		Item 11	I avoid patients with complex medical problems to prevent legal issues.	3.17	±1.21	0.669	0.918	0.925
		Item 12	I avoid treatment protocols with high complication rates to prevent legal problems.	3.31	±1.21	0.728	0.916	0.924
		Item 13	I prefer non-invasive treatment protocols instead of invasive ones to avoid legal issues.	3.25	±1.16	0.604	0.919	0.927
		Item 14	When malpractice-related topics are frequently discussed in the media, I feel uneasy about my medical practice.	3.77	±1.11	0.732	0.916	0.923
	Knowledge Level	Item 15	Have you ever been sued for malpractice during your medical career?	1.91	±1.34	0.206	0.93	0.934
		Item16	Do you think malpractice lawsuits affect physicians’ professional performance?	4.11	±1.2	0.618	0.919	0.926
		Item 17	Have you ever heard of the concept of defensive medicine?	3.47	±1.48	0.48	0.923	0.93
		Item 18	Do you think you have sufficient knowledge about the concept of defensive medicine?	2.66	±1.32	0.434	0.924	0.931

**Table 3.** The Comparison of the Surgical and Internal Sciences

	**Surgical Sciences (N = 110)**					**Internal Sciences (N = 152))**						**Covariate Analysis Result**	
	**Mean**	**SD**	**Median**	**Minimum**	**Maximum**	**Mean**	**SD**	**Median**	**Minimum**	**Maximum**	P*	How Long Have You Been Working as an Assistant? (Months)	What Is Your Average Weekly Working Hours? (Hours)
Age	30.10	4.53	30	4	42	31.10	5.65	30	24	52	.965		
Duration of residency (months)	36.00	19.71	36	1.5	62	31.50	17.86	30	1	72	.047		
Average weekly working hours	59.00	16.23	60	10	115	48.10	19.57	40	7	168	<.001		
Total score of Malpractice Fear Scale	22.20	5.85	23	6	30	23.60	4.82	24	6	30	.075	0.042	0.064
Positive defensive medicine practices	30.50	8.20	31	9	45	31.90	6.92	33	9	45	.224	0.203	0.285
Negative defensive medicine practices	16.60	5.33	15.5	5	25	17.10	4.77	17	5	25	.411	0.506	0.521
Knowledge level	12.20	4.06	12	4	20	12.10	3.27	12.5	4	20	.910	0.751	0.670
Total score of Defensive Medicine Attitude Scale	106.40	28.40	106	32	160	110.00	23.14	111.5	32	160	.283	0.335	0.412

**Table 4.** The Comparison of the Gender and Department

	**Department**			
Gender	**Surgical Sciences**	**Internal Sciences**	**χ^2^**	***P***
Male	64	82	0.464	.496
Female	46	70		
Total	110	152		

χ^2^, chi-square test.

**Table 5.** The Correlations Between the Scales

**Correlations**					
		**Positive Defensive Medicine Practices**	**Negative Defensive Medicine Practices**	**Knowledge Level**	**Total Score on the Defensive Medicine Practices Attitude Scale**
Total score on the Malpractice Fear Scale	r	0.69	0.574	0.449	0.695
	*P*	<.001	<.001	<.001	<.001
	N	262	262	262	262

**Table 6.** The Results of Structural Equation Models

**Indirect and Total Effects**									
**Model**	**Type**	**Effect**	**Estimate**	**SE**	**95% CI Lower**	**95% CI Upper**	***β***	**z**	***P***
Model 1	Indirect	Malpractice Fear Scale total score ⇒ How long have you been an assistant? (Months) ⇒ Defensive Practice Attitude Scale total score	0.004	0.021	−0.037	0.045	0.001	0.178	.859
	Component	Malpractice Fear Scale total score ⇒ How long have you been an assistant? (Months)	−0.343	0.217	−0.769	0.082	−0.097	−1.583	.113
		How long have you been a resident? (Months) ⇒ Defensive Practice Attitude Scale total score	−0.011	0.061	−0.130	0.108	−0.008	−0.179	.858
	Direct	Malpractice Fear Scale total score ⇒ Defensive Practice Attitude Scale total score	3.330	0.214	2.911	3.749	0.695	15.572	<.001
	Total	Malpractice Fear Scale total score ⇒ Defensive Practice Attitude Scale total score	3.334	0.213	2.916	3.752	0.695	15.633	<.001
Model 2	Indirect	Malpractice Fear Scale total score ⇒ What is your average weekly working hours? (Hours) ⇒ Defensive Practice Attitude Scale total score	0.002	0.020	−0.038	0.041	0.000	0.0837	.933
	Component	Malpractice Fear Scale total score ⇒ What is your average weekly working hours? (Hours)	−0.336	0.220	−0.767	0.095	−0.094	−1.5297	.126
		What is your average weekly working hours? (Hours) ⇒ Defensive Practice Attitude Scale total score	−0.005	0.060	−0.122	0.112	−0.004	−0.0838	.933
	Direct	Malpractice Fear Scale total score ⇒ Defensive Practice Attitude Scale total score	3.332	0.214	2.913	3.751	0.695	15.586	<.001
	Total	Malpractice Fear Scale total score ⇒ Defensive Practice Attitude Scale total score	3.334	0.213	2.916	3.752	0.695	15.633	<.001
Model 3	Indirect	Malpractice Fear Scale total score ⇒ What is your average weekly working hours? (Hours) ⇒ Positive defensive medicine practices	0.004	0.006	−0.009	0.016	0.003	0.586	.558
	Component	Malpractice Fear Scale total score ⇒ What is your average weekly working hours? (Hours)	−0.336	0.220	−0.767	0.095	−0.094	−1.53	.126
		What is your average weekly working hours? (Hours) ⇒ Positive defensive medicine practices	−0.011	0.018	−0.046	0.024	−0.028	−0.635	.525
	Direct	Malpractice Fear Scale total score ⇒ Positive defensive medicine practices	0.970	0.063	0.846	1.094	0.687	15.315	<.001
	Total	Malpractice Fear Scale total score ⇒ Positive defensive medicine practices	0.974	0.063	0.850	1.097	0.690	15.402	<.001
Model 4	Indirect	Malpractice Fear Scale total score ⇒ What is your average weekly working hours? (Hours) ⇒ Negative defensive medicine practices	−0.002	0.005	−0.011	0.007	−0.002	−0.422	.673
	Component	Malpractice Fear Scale total score ⇒ What is your average weekly working hours? (Hours)	−0.336	0.220	−0.767	0.095	−0.094	−1.53	.126
		What is your average weekly working hours? (Hours) ⇒ Negative defensive medicine practices	0.006	0.013	−0.020	0.032	0.022	0.439	.661
	Direct	Malpractice Fear Scale total score ⇒ Negative defensive medicine practices	0.543	0.048	0.449	0.637	0.576	11.34	<.001
	Total	Malpractice Fear Scale total score ⇒ Negative defensive medicine practices	0.541	0.048	0.447	0.634	0.574	11.323	<.001
Model 5	Indirect	Malpractice Fear Scale total score ⇒ What is your average weekly working hours? (Hours) ⇒ Knowledge level	−0.002	0.004	−0.009	0.005	−0.003	−0.512	.609
	Component	Malpractice Fear Scale total score ⇒ What is your average weekly working hours? (Hours)	−0.336	0.220	−0.767	0.095	−0.094	−1.53	.126
		What is your average weekly working hours? (Hours) ⇒ Knowledge level	0.006	0.011	−0.015	0.026	0.030	0.543	.587
	Direct	Malpractice Fear Scale total score ⇒ Knowledge level	0.307	0.038	0.233	0.381	0.452	8.157	<.001
	Total	Malpractice Fear Scale total score ⇒ Knowledge level	0.305	0.038	0.232	0.379	0.449	8.122	<.001

## Data Availability

The data that support the findings of this study are available on request from the corresponding author.
